# Serositis as an indicator of poor prognosis in pediatric systemic lupus erythematosus

**DOI:** 10.1186/s12969-025-01084-5

**Published:** 2025-04-01

**Authors:** Wei-Chen Kao, Ya-Chiao Hu, Jyh-Hong Lee, Li-Chieh Wang, Yu-Tsan Lin, Yao-Hsu Yang, Bor-Luen Chiang, Hsin-Hui Yu

**Affiliations:** 1 Department of Pediatrics, National Taiwan University Children’s Hospital, No. 7, Zhongshan S. Road, Zhongzheng District, Taipei City, 100 Taiwan; 2 Department of Pediatrics, Taipei Tzu Chi Hospital, Buddhist Tzu Chi Medical Foundation, New Taipei, Taiwan; 3 Department of Medical Research, National Taiwan University Hospital, Taipei, Taiwan; 4 Genome and Systems Biology Degree Program, College of Life Science, National Taiwan University, Taipei, Taiwan

**Keywords:** Childhood-onset systemic erythematous lupus (cSLE), Serositis, End-stage renal disease (ESRD), Survival, Children

## Abstract

**Background:**

Systemic lupus erythematosus (SLE) is a multi-systemic autoimmune disease that causes inflammation of the serosa (serositis). This retrospective study aimed to evaluate the clinical characteristics of serositis in childhood-onset SLE (cSLE) and analyze its association with long-term outcomes.

**Methods:**

We retrospectively reviewed the medical records of patients with cSLE diagnosed at a medical center in Taiwan, analyzing data collected from January 2002 to December 2022. We analyzed the clinical features of patients with serositis as pleuritis and/or pericarditis with at least a small effusion (> 0.5 cm in depth) on sonography or chest radiography. Cox proportional hazards regression was used to calculate the hazard ratios (HR) and 95% confidence intervals (CI) for the association between serositis and all-cause mortality.

**Results:**

185 patients with cSLE were enrolled, of whom 38 (20.54%) had serositis. Patients with serositis had a younger age at SLE diagnosis, a higher SLE Disease Activity Index 2000 score at serositis diagnosis, and an increased prevalence of lupus nephritis, central nervous system manifestations, end-stage renal disease (ESRD), a higher Systemic Lupus International Collaborating Clinics (SLICC)/American College of Rheumatology (ACR) damage index score, and a higher mortality than that of patients without serositis. Multivariate Cox regression analysis showed that both serositis (hazard ratio [HR]: 5.585, confidence interval [CI]: 1.853–17.80) and ESRD (HR: 13.956; CI: 3.822–50.964) were associated with mortality risk. Kaplan–Meier survival curve analysis revealed that patients with both serositis and ESRD had the poorest 20-year survival rate. Patients with late-onset serositis (occurring 1 year after SLE diagnosis) had higher mortality rates than those with early-onset serositis.

**Conclusion:**

Children with lupus serositis had higher disease activity, a higher prevalence of comorbidities, and mortality. Patients with both serositis, especially late-onset serositis, and ESRD had an increased risk of poor long-term survival.

**Supplementary Information:**

The online version contains supplementary material available at 10.1186/s12969-025-01084-5.

## Key message

• Patients with childhood-onset systemic erythematosus lupus (cSLE) and significant serositis exhibited high disease activity and an increased prevalence of lupus nephritis, central nervous system lupus, end-stage renal disease (ESRD), a higher Systemic Lupus International Collaborating Clinics (SLICC)/American College of Rheumatology (ACR) damage index score, and a higher mortality.

• Serositis and ESRD were significantly associated with an increased risk of mortality in patients with childhood-onset SLE.

• Patients with late-onset serositis that occurred one year after the diagnosis of SLE had significantly high mortality.

**Supplementary Information:**

The online version contains supplementary material available at 10.1186/s12969-025-01084-5.

## Introduction

Systemic lupus erythematosus (SLE) is a multisystem disease with a highly variable presentation and clinical course. Its etiology is multifactorial and complex, with all the key components of the immune system involved in the mechanism of autoimmunity and inflammation [1]. Deposition of immune complexes consisting of immunoglobulin G and complement and the subsequent inflammation of the serosa are considered to be involved in the pathogenesis of lupus serositis. The manifestations of serositis include pericarditis, pericardial effusion, pleuritis, pleural effusion, and peritonitis (ascites) in patients with SLE [2–4]. The incidence rate of serositis in patients with SLE is 33–80% [5, 6]. Pleural effusion and pericardial involvements are common; however, lupus peritonitis is rare [7]. While pleuritis and pericarditis account for four points in the SLE Disease Activity Index 2000 (SLEDAI-2 K), lupus peritonitis does not always correlate with lupus activity [8]. Pericarditis may range from a small, silent effusion to a massive effusion causing cardiac tamponade, a life-threatening condition.

Few studies have focused on the impact of lupus serositis on long-term outcomes, especially in childhood-onset SLE (cSLE). One study showed that lupus serositis responded well to non-steroidal anti-inflammatory drugs and corticosteroid treatment and had a favorable prognosis in adult patients with SLE [9]. Another study showed that adult patients with SLE and serositis were more likely to have lupus nephritis (LN), low complement levels, and high anti-double-stranded deoxyribonucleic acid (dsDNA) titers [10].

Compared with adult patients with SLE, cSLE has higher disease activity, a higher incidence of pericardial effusion, more rapid organ damage such as renal, neurological, and hematological involvement, and increased comorbidities [6, 11, 12]. In this single-center, retrospective, 20-year observational cohort study, we investigated the epidemiology and clinical characteristics of serositis in cSLE and analyzed the association between serositis and long-term outcomes, particularly mortality.

## Methods

### Study design

This retrospective observational study was conducted at the National Taiwan University Children’s Hospital. Eligible patients were diagnosed with SLE with onset before the age of 18 years between January 2002 and December 2022. Medical records of each patient from the outpatient department and during hospitalization were reviewed. The requirement for informed consent was waived for patients who underwent medical record review and anonymous clinical data analysis during the retrospective part of the study. This study was approved by the Institutional Research Ethics Committee (approval number: 201812007RIND).

SLE was diagnosed based on the classification criteria of the European Alliance of Associations for Rheumatology (EULAR), the American College of Rheumatology (ACR), or the Systemic Lupus International Collaborating Clinics (SLICC) [13–15]. Patient follow-up continued until December 31, 2022. Demographic data, including sex and age at the time of SLE diagnosis, laboratory data, and SLEDAI-2 K scores at the time of SLE diagnosis (in patients without serositis) or at the time of serositis diagnosis (in patients with serositis) were recorded. A modified SLEDAI-2 K score (SLEDAI-2 K scores minus the scores of serositis) was also calculated, which presented the non-serositis part of lupus activity. Admission to the pediatric intensive care unit (PICU), the usage of major treatment regimens, the prevalence of comorbidities (e.g. infections, ESRD), death, and the SLICC/ACR damage index score [16] at the end of study for each patient were recorded.

The diagnostic steps for serositis were based on both symptoms and routine procedures, including chest X-ray, electrocardiogram, cardiopulmonary ultrasound, or chest computerized tomography. Lupus pericarditis was suspected based on the presence of one or more of the following clinical manifestations: typical sharp precordial pains, pericardial rubs, cardiomegaly on chest radiography, or electrocardiographic abnormalities indicative of pericarditis. It was confirmed by demonstrating the presence of pericardial effusion (> 0.5 cm in depth) on an echocardiogram [9, 17]. Lupus pleuritis was suspected when dyspnea, pleuritic chest pain, or pleural effusion on chest radiography presented. It was confirmed by the presence of pleural effusion (> 0.5 cm in depth) on transthoracic sonography [9, 18, 19].

Infection-related pleuritis or pericarditis was diagnosed based on the cultures or polymerase chain reaction (PCR) results of pathogens (bacterial, fungal, mycobacterial, or viral) in the pleural or pericardial effusion, or it was highly suspected based on the treatment response to broad-spectrum antimicrobial agents. Lupus pericarditis or pleuritis was diagnosed by the clinical and laboratory parameters of lupus activity and the judgment of pediatric rheumatologists. Trivial pericardial and pleural effusions (< 0.5 cm in depth), or effusions due to other conditions, such as infections, malignancy, or fluid overload, were excluded in the analysis.

We further classified patients with either early-onset serositis (serositis developed within one year of the SLE diagnosis) or late-onset serositis (serositis developed more than one year after SLE diagnosis) according to the interval from SLE diagnosis to serositis. The amount of serositis (small, moderate, and large amount) by the volume of the effusion was recorded [17–19]. Patients were classified as having moderate-to-severe or small amount of serositis in the subgroup analysis.

In accordance with EULAR/ACR classification criteria [15], hematological manifestations were defined as white cell count < 4 × 10^9^/L, platelet count < 100 × 10^9^/L, or evidence of autoimmune hemolysis. Renal involvement was defined as hematuria, proteinuria > 0.5 g/24 hours, or lupus nephritis confirmed by renal biopsy according to the International Society of Nephrology/Renal Pathology Society (ISN/RPS) 2003 classification [20]. End-stage renal disease (ESRD) was defined as a glomerular filtration rate of less than 15 ml/min/1.73m^2^ for more than 3 months [21]. Neuropsychiatric manifestations were based on the nomenclature and standard definitions for neuropsychiatric SLE (NPSLE) established by the ACR [22].

### Statistical analysis

The variables from each category were reported as numbers (n) and percentages (%), and continuous data were presented as means with standard deviation or medians with ranges. The chi-square test was used to compare categorical data between the two groups. The Student’s t-test, or Mann–Whitney U test, was used to compare numerical data. In addition, multivariate Cox proportional hazards regression was used to calculate the hazard ratio (HR) and 95% confidence intervals (CI) for the association between serositis and mortality. Sex, diagnostic age of SLE, ESRD, and a modified SLEDAI-2 K score (in categories of > 8 or ≤ 8 points) as potential confounders were included in this model. To enhance the possibility of comparison between patients with and without serositis, a modified SLEDAI-2 K score was used. Cumulative survival probability was evaluated using the Kaplan–Meier survival analysis.

Statistical computations and the generation of graphical representations were performed using GraphPad (version 8.0.2 for Windows; GraphPad Software, La Jolla, CA, USA) and SPSS software (version 29.0 for Windows; IBM Corp., Armonk, NY, USA). All statistical tests were two-sided, and a *p*-value < 0.05 was considered significant.

## Results

We enrolled 185 patients with cSLE, and 38 (20.54%) patients were diagnosed with lupus serositis. Table 1 listed the demographic, clinical manifestations, laboratory data, main treatment, comorbidities, and outcomes. The mean age at SLE diagnosis in patients with cSLE with serositis was significantly younger than that in patients without serositis (12.52 ± 3.13 vs. 13.69 ± 3.13 years, *p* = 0.041). The mean age at serositis diagnosis was 15.19 ± 5.11 years. The disease activity SLEDAI-2 K score and modified SLEDAI-2 K in patients with cSLE with serositis were significantly higher than that of those in patients with cSLE without serositis. Patients with cSLE who had serositis had a higher prevalence of LN, CNS lupus, ESRD, and a higher rate of PICU admissions than those who did not have serositis over the course of the whole follow-up period. A higher percentage of patients with serositis received cyclophosphamide pulse therapy than that of those without serositis (42.1% vs. 14.28%, *p* < 0.001). We observed a higher SLICC/ACR damage index score in patients with cSLE with serositis compared to patients with cSLE without serositis at the study end-point (2.05 ± 1.65 vs. 0.26 ± 0.77, *p* < 0.001) (Table 1). During a median follow-up of 8.74 ± 6.16 years, the mortality rate of all patients was 10.81%. Patients with serositis had a higher mortality rate than those without serositis (39.47% vs. 3.40%, *p* < 0.001).

**Table 1:** Demographic data of patients with childhood-onset systemic lupus erythematosus (SLE) with and without serositis

	All patients (*N* = 185)	cSLE with serositis (*N* = 38, 20.54%)	cSLE without serositis (*N* = 147, 79.45%)	*P*-value
Female: Male (% of females)	155:30 (83.78%)	33:5 (86.84%)	122:25 (82.99%)	0.743
Age at diagnosis of SLE (years)	13.45 ± 3.17	12.52 ± 3.13	13.69 ± 3.13	**0.041**
Follow-up duration (years)	8.74 ± 6.16	7.02 ± 6.01	9.19 ± 6.12	0.053
SLEDAI-2 K score^a^	11.78 ± 5.40	17.86 ± 4.80	10.21 ± 4.32	**< 0.001**
Modified SLEDAI-2 K score^a^	11.10 ± 4.80	14.55 ± 4.98	10.21 ± 4.32	**< 0.001**
Laboratory data^b^		(at onset of serositis)	(at SLE diagnosis)	
White blood cells (x10^9^/L)	6.11 ± 4.54	7.72 ± 5.62	5.68 ± 4.09	**0.013**
C3 (mg/dL)	52.65 ± 29.2	46.57 ± 27.68	54.29 ± 29.38	0.15
C4 (mg/dL)	9.29 ± 7.32	9.31 ± 6.42	9.29 ± 7.55	0.986
Anti-dsDNA (IU/ml)	568.29 ± 430.26	547.52 ± 492.38	573.88 ± 411.74	0.739
Lupus manifestations^c^				
Lupus nephritis	95 (51.35%)	31 (81.54%)	64 (44.89%)	**< 0.001**
CNS manifestations	19 (10.27%)	14 (36.84%)	5 (3.40%)	**< 0.001**
Hematological involvement	90 (47.6%)	23 (60.52%)	63 (42.85%)	0.077
Treatment^c^				
Mycophenolic acid^d^	146 (78.91%)	25 (65.78%)	121 (82.8%)	**0.045**
Rituximab^e^	72 (38.91%)	12 (31.57%)	60 (41.4%)	0.392
Methylprednisolone pulse therapy	120 (64.86%)	30 (78.94%)	90 (62.6%)	0.064
Cyclophosphamide pulse therapy	37 (20%)	16 (42.1%)	21 (14.28%)	**< 0.001**
PICU admission	34 (18.37%)	23 (60.52%)	11 (7.48%)	**< 0.001**
Comorbidities and outcomes^c^				
End-stage renal disease	28 (14.8%)	18 (47.36%)	10 (6.80%)	**< 0.001**
SLICC/ACR damage index score	0.63 ± 1.25	2.05 ± 1.65	0.26 ± 0.77	**< 0.001**
Mortality	20 (10.81%)	15 (39.47%)	5 (3.40%)	**< 0.001**
Lost to follow-up	34 (18.37%)	8 (21.05%)	26 (17.68%)	0.808

Abbreviations: cSLE, childhood-onset systemic lupus erythematosus; dsDNA, double-stranded deoxyribonucleic acid; PICU, pediatric intensive care unit; SLE, systemic lupus erythematosus; SLEDAI-2 K, Systemic Lupus Erythematosus Disease Activity Index 2000; CNS, central nervous system; SLICC/ACR, Systemic Lupus International Collaborating Clinics/American College of Rheumatology

Data are expressed as number of patients (%) or mean ± standard deviation (SD)

^a^Modified SLEDAI-2 K score: SLEDAI-2 K scores minus scores of serositis; SLEDAI-2 K score or modified SLEDAI-2 K score were recorded at the diagnosis of serositis (patients with serositis) or SLE (patients without serositis)

^b^Normal ranges: white blood cells: 3.84–11.40 (x10^9^/L); C3: 87–200 (mg/dL); C4: 10–52 (mg/dL); anti-dsDNA antibodies: negative ≤ 200, equivocal 201–300, positive ≥ 301 (IU/ml)

^c^Prevalence of events or medication usage from the diagnosis of SLE to study endpoints

^d^Mycophenolic acid or mycophenolate mofetil

^e^Rituximab 375 mg/m^2^ or up to 500 mg per dose

In Table 2, we classified patients with serositis as either early-onset (*N* = 19, 50%) or late-onset (*N* = 19, 50%). In addition, 12 (31.57%) patients presented with serositis simultaneously with the SLE diagnosis. Patients with late-onset serositis had a higher prevalence of ESRD (73.68% vs. 21.05%, *p* = 0.003) and death (57.89% vs. 21.05%, *p* = 0.046) than that of those with early-onset serositis. Among these cSLE patients with serositis, 23 (60.52%) patients had small amount of effusion, 9 (23.68%) patients had moderate amount of effusion, and 6 (15.78%) patients had large amount of effusion. Patients having moderate-to-large serositis did not significantly differ in the clinical outcomes in comorbidities or mortality when comparing patients with small amount of serositis (Table 3).

**Table 2:** Comparison between patients with childhood-onset SLE with early- and late-onset serositis

	cSLE with early-onset serositis (*N* = 19, 50%)	cSLE with late-onset serositis (*N* = 19, 50%)	*P*-value
Female: Male, n (%)	16:3 (84.21%)	17:2 (89.47%)	1.000
Age at diagnosis of SLE, mean ± SD, years	12.87 ± 3.59	12.17 ± 2.54	0.507
Follow-up duration, mean ± SD, years	4.33 ± 4.99	9.70 ± 5.73	0.004
Modified SLEDAI-2 K, mean ± SD	15.52 ± 5.18	13.57 ± 4.58	0.240
Lupus nephritis, n (%)	13 (68.42%)	18 (94.73%)	0.094
End-stage renal disease, n (%)	4 (21.05%)	14 (73.68%)	**0.003**
Mortality, n (%)	4 (21.05%)	11 (57.89%)	**0.046**
Age at death, mean ± SD, years	14.25 ± 2.46	18.04 ± 4.80	0.183

cSLE, childhood-onset systemic lupus erythematosus; SLE, systemic lupus erythematosus; SD, standard deviation; SLEDAI-2 K, Systemic Lupus Erythematosus Disease Activity Index 2000

**Table 3:** Comparison of childhood-onset systemic lupus erythematosus (SLE) according to serositis severity

	cSLE with large and moderate serositis (*N* = 15, 39.47%)	cSLE with small serositis (*N* = 23, 60.52%)	*P*-value
Female: Male, n (%)	14:1 (93.3%)	19:4 (82.60%)	0.641
Age at diagnosis of SLE, mean ± SD, years	13.40 ± 2.18	11.94 ± 3.50	0.168
Follow-up duration, mean ± SD, years	5.82 ± 6.46	7.80 ± 5.56	0.344
Performance of effusion drainage, n (%)	10 (66.66%)	6 (26.08%)	**0.032**
Modified SLEDAI-2 K, mean ± SD	14.20 ± 4.95	14.78 ± 4.99	0.733
Lupus nephritis, n (%)	12 (80%)	19 (82.60%)	0.821
End-stage renal disease, n (%)	9 (60%)	9 (39.13%)	0.353
Mortality, n (%)	7 (46.66%)	8 (34.78%)	0.694
Age at death, mean ± SD, years	15.13 ± 2.49	18.69 ± 5.35	0.156

cSLE, childhood-onset systemic lupus erythematosus; SLE, systemic lupus erythematosus; SD, standard deviation; SLEDAI-2 K, Systemic Lupus Erythematosus Disease Activity Index 2000

In patients with serositis who died (*N* = 15, female: male 13:2), the median time from the onset of serositis to death was 99 days (range from 7 to 353 days), and 10 (66.66%) of these 15 patients expired within 6 months after serositis onset (Supplementary Table S1). Lupus manifestations or comorbidities that complicated the course of serositis included infections (100%), ESRD (73.33%), central nervous system vasculitis (46.66%), hematologic crisis (26.66%), and macrophage activation syndrome (MAS) (20%) in these 15 patients. The direct causes of death were septic shock (*N* = 9), intracranial hemorrhage (ICH) (*N* = 5), and cardiac tamponade (*N* = 1). Among infection episodes (including sepsis/septic shock, pneumonia, infective endocarditis, and urinary tract infection), the three most common pathogens identified were *Stenotrophomonas maltophilia (26.66%)*,* Klebsiella pneumoniae (26.66%)*, and *Acinetobacter baumannii (20%)*. Opportunistic pathogens were also identified, such as *Aspergillus*,* Candida albicans*, and *Pneumocystis jirovecii.*

**Fig. 1:**
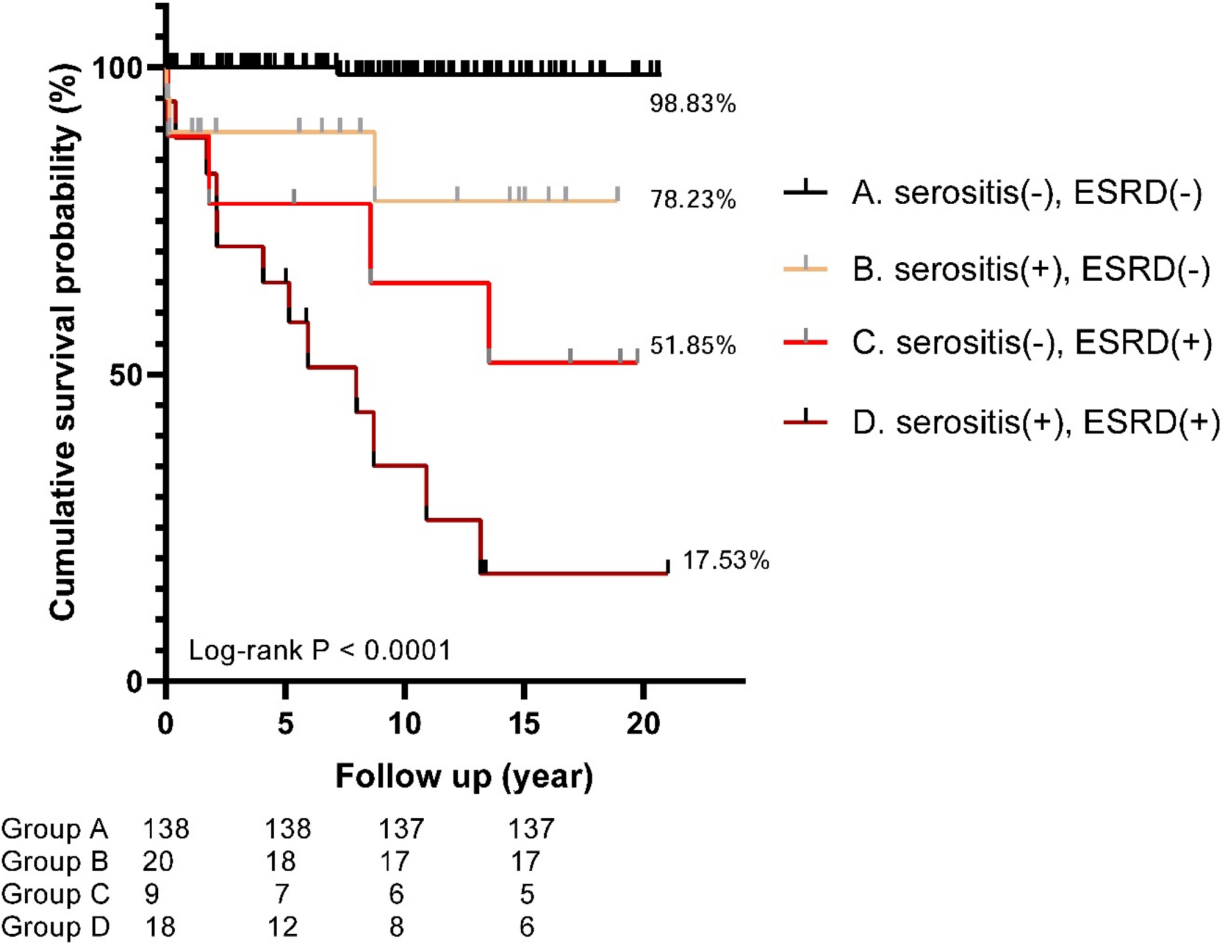
Kaplan–Meier survival analysis shows the cumulative survival probabilities of patients with childhood-onset SLE divided by with and without serositis or end-stage renal disease ESRD, end-stage renal disease

In Table 4, multivariate Cox regression analysis revealed that serositis (HR: 5.585, CI: 1.853–17.80) and ESRD (HR: 13.956; CI: 3.822–50.964) were significantly associated with a higher risk of death after adjusting for sex, age at SLE diagnosis, and the modified SLEDAI-2 K score. We further analyzed survival probabilities based on the presence or absence of serositis and ESRD using Kaplan–Meier curves (Fig. 1). The estimated 5-year, 10-year, and 15-year survival probabilities were 93.24%, 87.53%, and 82.28% for all patients in our cohort. The estimated 15-year survival probabilities were 98.83% for patients without serositis or ESRD, 78.23% for those with serositis but without ESRD, 51.85% for those without serositis but with ESRD, and 17.53% for those with both serositis and ESRD, respectively (log-rank test, *p* < 0.0001).

**Table 4:** Risk of death in patients with childhood-onset SLE by multivariate Cox regression analysis

	cSLE deceased (*N* = 20, 10.81%)	cSLE survivors (*N* = 165, 89.18%)	Hazard ratio	95% Confidence interval	*P*-value
Female, n (%)	18 (90%)	137 (83.03%)	1.830	0.330–10.166	0.48
Age at diagnosis of SLE (years)	12.66 ± 2.80	13.55 ± 3.20	1.023	0.868–1.205	0.787
Serositis, n (%)	15 (75%)	23 (13.93%)	5.585	1.853–17.80	**0.003**
End-stage renal disease, n (%)	16 (84.21%)	12 (7.27%)	13.956	3.822–50.964	**< 0.001**
Modified SLEDAI-2 K > 8, n (%)	15 (75%)	108 (65.45%)	0.539	0.156–1.863	0.328

Age is expressed as mean ± SD

cSLE, childhood-onset systemic lupus erythematosus; SLE, systemic lupus erythematosus; SLEDAI-2 K, Systemic Lupus Erythematosus Disease Activity Index 2000

## Discussion

Our study analyzed the clinical features and long-term outcomes of 38 patients with cSLE with serositis (20.54%) from a cohort of 185 patients with cSLE. Pediatric patients with SLE with serositis had a younger age at SLE diagnosis, higher disease activity during serositis diagnosis, a higher rate of PICU admission, a higher prevalence of LN, CNS lupus, ESRD, and a higher SLICC/ACR damage index score than that of patients without serositis. The patients with cSLE with serositis also had low complement levels and high anti-dsDNA titers, indicating high disease activity during the presence of lupus serositis. Our results are consistent with those of previous studies showing higher disease activity in adult patients with SLE with serositis [6, 23]. In contrast to that in adult patients, we did not observe a significant association between serositis and cardiovascular events in patients with cSLE [10].

Although pleuritis and pericarditis are common cardiac and pulmonary manifestations of SLE, the primary diagnostic priority in our patients was to exclude infection, especially pneumonia or systemic infections. The combination of antinuclear antibodies (ANA) positivity (titer ≥ 1:80) and decreased C3 and C4 levels in pleural effusions demonstrated a sensitivity of 82%, specificity of 89%, and a negative predictive value of 93% for distinguishing lupus pleuritis from non-lupus exudative pleural effusion [24, 25]. In contrast, adenosine deaminase (ADA) activity was significantly elevated in infection-related pleural effusion [24]. A study of 2390 patients with SLE demonstrated that hemolytic anemia, proteinuria, lymphadenopathy, and anti-Sm antibodies were associated with pericarditis, while anti-DNA antibodies were associated with both pericarditis and pleuritis [23]. Pleuritis was also found to predict later gastrointestinal infarction or resection and was associated with long-term organ damage in SLE [23]. In our cohort, 31.57% of our patients with cSLE having serositis were diagnosed with serositis at the time of their SLE diagnosis, aligning with findings from previous studies comparing pericardial manifestation in adult- and childhood-onset SLE [26]. Additionally, half of our patients developed serositis within 1 year of their SLE diagnosis.

While adult patients with SLE with serositis receive increased methylprednisolone pulse therapy and higher maintenance doses of glucocorticoids [27], a higher percentage of our pediatric patients with lupus serositis received cyclophosphamide and methylprednisolone pulse therapy for high disease activity or deteriorating renal function due to LN. Notably, 72 (38.91%) patients with cSLE in our cohort received rituximab, and the percentage of rituximab use was similar in patients with and without serositis. The use of rituximab as an add-on maintenance therapy for LN has been shown to decrease the cumulative maintenance dose of glucocorticoids and achieve more favorable LN control according to our previous study on cSLE [28].

Despite aggressive treatment, significant comorbidities, and death (39.47%) occur in patients with serositis. In patients with cSLE with serositis who expired, the median time from serositis diagnosis to death was 99 days. One-third of these patients died 1 month after the onset of serositis, and 66.66% died within 6 months after serositis onset. Septic shock and ICH are leading causes of death in patients with serositis. In addition to Gram-negative bacteria-related sepsis, fungal and *Pneumocystis jirovecii* opportunistic infections also contribute to mortality, suggesting a fulminant course due to the immunocompromised status. The risk factors for severe infections, ICH, and high disease activity in our patients with serositis included bone marrow suppression after immunosuppressant use and prolonged leukopenia, lymphopenia, thrombocytopenia, and ESRD. In contrast to our previous study on cSLE, which showed that the most common form of CNS lupus was ischemic stroke [29], the current study showed that ICH was more common in patients with cSLE and serositis. Increased incidence of ICH may be related to the combined effects of systemic inflammation, thrombocytopenia, high lupus activity, and severe infection in patients with SLE [30].

A recent study showed that the 10-year survival rates were 90.2–93.2% for adult SLE and 98.9–100% for cSLE in a medical center in Taiwan [31]. However, there is a high percentage of loss to follow-up in this retrospective study. Our previous study showed a mortality rate of 27% of all cSLE patients from 1985 to 2005 in our hospital [29]. In this study, we further showed that the overall mortality of patients with cSLE had much improvement to 10.81% in the recent 20 years.

Our study demonstrated that lupus serositis, particularly in the late-onset group, and ESRD are significantly associated with mortality, consistent with Chen et al.‘s study on pericarditis in adult SLE [32]. Previous studies have shown fibrinous pericarditis with immunoglobulin, C1q, and C3 deposition in the walls of the blood vessels of the myocardium and pericardium, as observed through direct immunofluorescence [33–35]. Severe inflammation and immune complex-mediated injury in myocardial and pericardial tissues, as noted in autopsy findings, correlated with marked clinical and serological disease activity in SLE [34]. Pericarditis in SLE can present as a chronic process, an isolated attack, or recurrent brief episodes. The development of heart failure is often associated with pericarditis, hypertension, fluid retention due to renal disease, or corticosteroid use [36]. In addition to ESRD, which has a significant impact on the survival of patients with both adult- and childhood-onset SLE [37, 38], other poor prognostic factors for mortality include higher daily doses of corticosteroid, chronic kidney disease, and severe infections [31]. Previous studies on adult SLE showing a favorable prognosis for SLE-related serositis primarily focused on serositis occurring at or shortly after the diagnosis of SLE, similar to the patients with early-onset serositis in our study [9, 39].

Our study had several strengths, including the accuracy of SLE diagnosis and the inclusion of data on comorbidities, causes of death, and factors associated with survival. Our study also had some limitations, including a small sample size and a retrospective study design. We may have only reported serositis with effusion more than 0.5 cm in depth in a medical center, which potentially limits the generalizability of our results to the entire cohort. The findings of this study should enhance the clinicians’ awareness of the negative impact of serositis on cSLE outcomes.

## Conclusion

In summary, our study of a cSLE cohort showed that patients with serositis had higher disease activity, increased prevalence of comorbidities, a higher SLICC/ACR damage index score, and a higher mortality. Patients with both serositis, especially late-onset serositis, and ESRD had a significantly increased risk of mortality and poor long-term survival.

## Electronic supplementary material

Below is the link to the electronic supplementary material.

Supplementary Material 1

## Data Availability

No datasets were generated or analysed during the current study.

## Abbreviations

ANA: Antinuclear antibodies
cSLE: Childhood-onset SLE
dsDNA: Double-stranded deoxyribonucleic acid
ESRD: End-stage renal disease
ICH: Intracranial hemorrhage
LN: Lupus nephritis
MAS: Macrophage activation syndrome
NPSLE: Neuropsychiatric SLE
PCR: Polymerase chain reaction
SLE: Systemic lupus erythematosus
