# Supplementary material for: Serositis as an indicator of poor prognosis in pediatric systemic lupus erythematosus
Source: Pediatr Rheumatol Online J. 2025 Apr 1;23:36. doi: 10.1186/s12969-025-01084-5 (PMC11959889; doi:10.1186/s12969-025-01084-5)
Supplement: Supplementary file 1 — Supplementary Material 1 [file 12969_2025_1084_MOESM1_ESM.pdf]

Supplementary Table S1. Clinical characteristics of patients with childhood-onset SLE with serositis who died (N = 15).

Characteristics	
Female, n (%)	13/15 (86.66%)
Age of death, mean ± SD, years	17.03 ± 4.61
Interval between serositis and mortality	
Median (range), days	99 (7-353)
Less than 1 month, n (%)	5 (33.33%)
Less than 6 months, n (%)	10 (66.66%)
Lupus manifestations or comorbidities complicated the course within 6 months since serositis	
Infections	15 (100%)
End-stage renal disease	11 (73.33%)
Central nervous system vasculitis	7 (46.66%)
Hematologic crisis	4 (26.66%)
Macrophage activation syndrome	3 (20%)
Cause of mortality, n (%)	
Septic shock	9 (60%)
Intracranial hemorrhage	5 (33.33%)
Cardiac tamponade	1 (6.66%)

Pathogen cultured from expired patients, n (%)	
Stenotrophomonas maltophilia	4 (26.66%)
Klebsiella pneumoniae	4 (26.66%)
Acinetobacter baumannii	3 (20%)
Aspergillus	3 (20%)
Candida albicans	3 (20%)
Pseudomonas aeruginosa	2 (13.33%)
Pneumocystis jirovecii	1 (6.66%)
Salmonella	1 (6.66%)
Staphylococcus aureus	1 (6.66%)

SD, standard deviation
